# Advances in the Applications of Polyhydroxyalkanoate Nanoparticles for Novel Drug Delivery System

**DOI:** 10.1155/2013/581684

**Published:** 2013-07-24

**Authors:** Anupama Shrivastav, Hae-Yeong Kim, Young-Rok Kim

**Affiliations:** Institute of Life Sciences and Resources & Department of Food Science and Biotechnology, College of Life Sciences, Kyung Hee University, Yongin 446-701, Republic of Korea

## Abstract

Drug delivery technology is emerging as an interdisciplinary science aimed at improving human health. The controlled delivery of pharmacologically active agents to the specific site of action at the therapeutically optimal rate and dose regimen has been a major goal in designing drug delivery systems. Over the past few decades, there has been considerable interest in developing biodegradable drug carriers as effective drug delivery systems. Polymeric materials from natural sources play an important role in controlled release of drug at a particular site. Polyhydroxyalkanoates, due to their origin from natural sources, are given attention as candidates for drug delivery materials. Biodegradable and biocompatible polyhydroxyalkanoates are linear polyesters produced by microorganisms under unbalanced growth conditions, which have emerged as potential polymers for use as biomedical materials for drug delivery due to their unique physiochemical and mechanical properties. This review summarizes many of the key findings in the applications of polyhydroxyalkanoates and polyhydroxyalkanoate nanoparticles for drug delivery system.

## 1. Introduction

Drug deliveries have become important tools in the medical field and have been extensively investigated because of the strong demand for the controlled delivery of pharmacologically active materials to cells, tissue, and organs. Many drug-delivery methods have been developed using polymers as drug carriers as they can effectively deliver the drug to a target site and thus increase the therapeutic benefit, while minimizing side effects. Because of the flexibility of polymers, it becomes possible to engineer multiple functionalities required for efficient drug delivery, simultaneously maintaining biocompatibility, facile manufacturing, and stable formulation. It is important that the biomaterial should complete the requirement of physical properties, but it is also essential to accomplish its biocompatibility test. Some of the traditionally used polymers like silicone have been suspected to cause cancer [1]. Therefore, there is a need for nontoxic, biodegradable, and biocompatible polymers.

**Figure 1:**
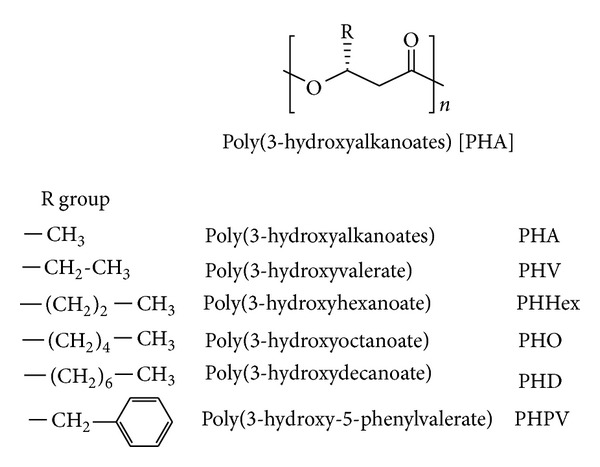
General structure of polyhydroxyalkanoates (PHAs) and examples of their structural derivatives.

 One of the alternatives to conventional polymers is biodegradable plastic or biopolymer. Among the various biodegradable polymers available, there is growing interest in the group of biopolymers known as polyhydroxyalkanoates (PHAs) which are the polyesters of various hydroxyalkanoate monomers accumulating as energy/carbon storage materials by granular inclusions in the cytoplasm of various bacterial cells, usually under unbalanced growth conditions. General structure of PHA is shown in Figure 1. There are approximately 150 different types of hydroxyalkanoic acids at present known as the constituents of these bacterial storage polyesters.

Bacterial PHAs could be bifurcated into two groups depending on the number of carbon atoms in the monomeric units: short-chain-length (SCL) PHAs, which consist of 3–5 carbon atoms, and medium-chain-length (MCL) PHAs, which consist of 6–14 carbon atoms [2]. PHAs are hydrophobic and crystalline in nature. Biologically produced PHAs are composed only of chirally pure (R)-configuration monomers. Beijerinck first observed PHAs as refractile bodies inside bacterial cells in 1888. However, the PHA composition was established by Lemoigne in 1926. Bacterial polyhydroxyalkanoates (PHAs) have attracted much attention as environmentally degradable thermoplastics [3, 4]. They are being viewed as potentially useful for replacing many synthetic polymers in a wide range of agriculture, marine, and medical applications.

The PHA polymers are stored in the bacterial cells as defined granules. These granular particles consist of polyester, proteins, and lipids. The composition was for the first time investigated by Griebel in 1968 for PHB granules of *Bacillus megaterium*, which consist of 97.7% of polyester, 1.87% of proteins, and 0.46% of lipids or phospholipids [5].

Particular attention has been focused on the use of poly(3-hydroxybutyrate) (PHB) and related copolymers, mostly poly(3-hydroxybutyrate-*co*-3-hydroxyvalerate) (PHBV), as carriers for drug delivery or scaffolds in tissue engineering. PHB and PHBV have many advantages when compared to other chemically produced polymers like polyglycolate, polylactate, and poly(lactide-co-glycolide) which include excellent biocompatibility, biodegradability, easier processibility, and the controllable retarding properties which can be modulated by variations in processing and molecular weight of the polymer composition. PHB in combination with other biocompatible and nontoxic polymers would also have an enhanced scope in biomedical applications [6]. The main advantage of using PHA in the medical field is that it is biodegradable and can be inserted into the human body, and it does not have to be removed again. Another distinguished characteristic of PHA is that it is biocompatible, generating a mild foreign-body response to any implant.

PHAs have been in the attention of many companies as biodegradable and biocompatible alternatives to synthetic polymers for a very long time. In 1976, Imperial Chemical Industries (ICI Ltd., UK) recognized the potential applicability of PHB to replace some of the oil-derived synthetic polymers. One of the contributions of PHA to medicine has been in the cardiovascular area. Tepha Inc., based in Cambridge, MA, has been devoted to manufacturing pericardial patches, artery augments, cardiological stents, vascular grafts, heart valves, implants, tablets, sutures, dressings, dusting powders, prodrugs, and microparticulate carriers using PHA. The first PHA-based product approved by FDA for clinical application is the TephaFLEX absorbable suture prepared from P4HB (http://www.tepha.com/). In 2007, the FDA had cleared its marketing in the USA, indicating a bright future for a practical application of PHAs in biomedical areas [7].

## 2. PHA Biosynthesis

PHAs are synthesized by varieties of Gram-positive and Gram-negative bacteria, and more than 300 different microorganisms are known to synthesize and accumulate PHAs intracellularly including *Azotobacter *sp., *Pseudomonas *sp., *Bacillus *sp., and *Methylobacterium *sp. [8]. These types of microbes are carrying metabolic ability to biosynthesize PHAs molecules and accumulate them in their cytoplasm as carbon and energy sources in the shape of granules under nutrient-limiting conditions with excess carbon [9, 10].

**Figure 2:**
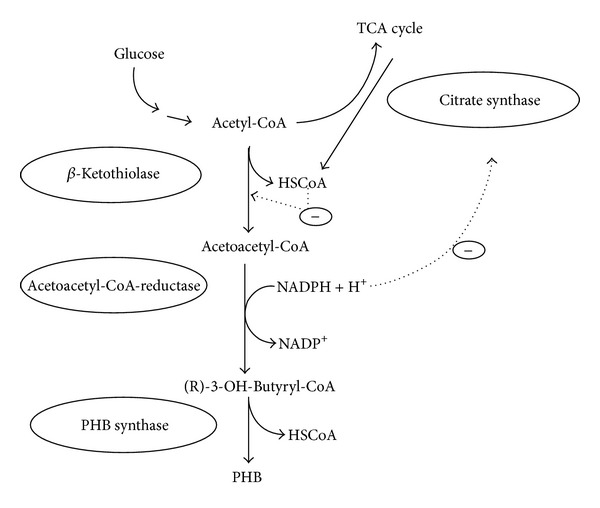
PHB synthesis pathway of *R. eutropha* and regulatory circuits. In the three-step PHB synthesis pathway, two acetyl-CoA molecules are coupled to form acetoacetyl-CoA in a condensation reaction catalysed by *β*-ketothiolase. The *β*-ketothiolase is negatively regulated by the product coenzyme A (HSCoA), which is also a product when acetyl-CoA enters the TCA cycle under nonlimited conditions. The product is subsequently and stereoselectively reduced to (R)-3-hydroxybutyryl-CoA in a reaction catalysed by NADPH-dependent acetoacetyl-CoA reductase. High concentration of NADPH and NADH inhibits the citrate synthase of the TCA cycle, which ensures the availability of acetyl-CoA for the *β*-ketothiolase. Finally, PHB is synthesized by polymerization of (R)-3-hydroxybutyryl-CoA molecules by the PHB synthase. Hatched arrows indicate negative regulatory effects. Reprinted with permission from Kessler and Witholt [11], Copyright (2001), Elsevier.

**Figure 3:**
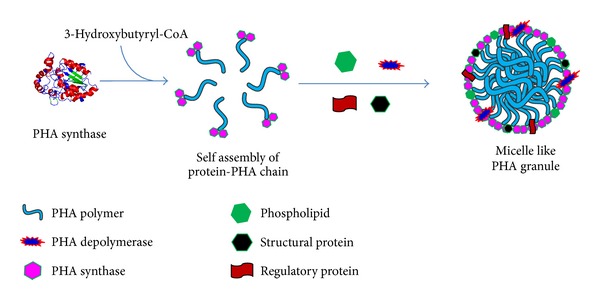
Schematic presentation of PHB granule formation within the cytoplasm of PHB producing microorganism. PHB is synthesized by polymerization of (R)-3-hydroxybutyryl-CoA molecules by PHB synthase leading to the formation of PHB granules. PHB inclusions consist of a hydrophobic core of amorphous PHB surrounded by phospholipid and various proteins.

The comprehensive investigation was done for metabolic biosynthetic pathways for PHA synthesis in *Cupriavidus necator* (formerly known as *Ralstonia eutropha*). This pathway is common in a wide range of bacteria. A *β*-ketothiolase catalyzes the formation of a carbon-carbon bond of two acetyl-CoA moieties. NADPH-dependent acetoacetyl-CoA reductase catalyzes the reduction of acetoacetyl-CoA formed in the first reaction to 3-hydroxybutyryl-CoA (Figure 2) [11]. PHB is synthesized by polymerization of (R)-3-hydroxybutyryl-CoA molecules by the PHB synthase leading to the formation of PHB granules (Figure 3). Two moles of acetyl-CoA are used to form an HB unit of the polymer, while an HV unit is formed by the reaction of acetyl CoA and propionyl-CoA [12].

PHA synthesis is also affected by the ratio of NADH to NAD^+^. CoA level in the cells is high during cell growth due to the rapid flux of acetyl-CoA into the tricarboxylic acid (TCA) cycle. When a nutrient, such as the nitrogen source, is exhausted, there is increase in the NADH/NAD^+^ ratio which inhibits the enzymes of the TCA cycle. As the flux of acetyl-CoA decreases, CoA levels decrease, removing the inhibition of *β*-ketothiolase. Acetyl-CoA or propionyl-CoA may enter the PHA biosynthetic pathway to produce 3HB or 3HV monomers [13, 14].

*Cupriavidus necator* has been the most commonly used strain for the industrial production of poly-(R)-3-hydroxybutyrate (PHB), poly((R)-3-hydroxybutyrate-*co*-4-hydroxybutyrate) (P3HB4HB), and poly((R)-3-hydroxybutyrate-*co*-(R)-3-hydroxyvalerate) (PHBV). Other bacteria including *Alcaligenes latus, Aeromonas hydrophila, Pseudomonas oleovorans, Pseudomonas putida,* and recombinant *Escherichia coli* are also used for PHV and PHBV production.

PHAs can be produced from recombinant *E. coli* by heterologously expressing the required PHB biosynthesis genes while providing appropriate cultivation conditions. PHA production in plants has also been taken into consideration. Recently, native PHA granules and *in vitro* synthesized PHA granules have been increasingly considered for applications as functionalized micro- or nanoparticles in biotechnology and biomedicine.

## 3. Biodegradability of PHA

One of the unique properties of biological PHA materials is their biodegradability in various environments. PHAs are of biological origin; they could be completely broken down in to water and carbon dioxide by microorganisms found in a wide range of environments, such as soil, water, and sewage [16]. A number of microorganisms such as bacteria and fungi in soil, sludge, and sea water excrete extracellular PHA degrading enzymes to hydrolyze solid PHA into water soluble oligomers and monomers and subsequently utilize the resulting products as nutrients within cells. The hydrolytic and enzymatic degradation processes of P(3HB-*co*-4HB) films were studied by monitoring the time-dependent changes in molecular weights and weight loss. P(3HB-co-4HB) films were shown to be hydrolyzed by both PHA depolymerase and lipase [17–19].

Pişkin reported that the deprivation of PHB *in vivo* is faster than *in vitro* hydrolysis at body temperature, indicating that enzymes existing *in vivo* catalyze the degradation [20]. *In vivo* degradation of P(3HB-*co*-4HB) films in rats was studied wherein P(3HB-*co*-4HB) films were implanted into the intraperitoneal area of rat, and changes in molecular weights were monitored over a period of 4 months. A 20% decrease in the value of Mn was observed with no notable signs of cytotoxicity in the implantation area [19]. PHA implants and other medical devices are degraded at the site of implantation in animals. PHA polymers are degraded by the action of nonspecific lipases and esterases in nature [21]. Löbler et al. detected lipase activities in the rat gastrointestine near the PHA implant, suggesting the involvement of lipases in the metabolism of PHA *in vivo* [22].

Degradation of PHA matrices in the tissues of the host organism offers the possibility of coupling this phenomenon with release of bioactive compounds, such as antibiotic or antitumor drug. If a PHA insert is impregnated with a compound, the degradation over time will release the compound, acting as an automatic dosing agent. The kinetics of dosing of a compound from a PHA matrix can be tuned by altering the polymer properties, along with the use of different types of PHA with different monomer side chains.

## 4. Biocompatibility of PHA

The suitability of PHA for inclusion in drug delivery or other biomedical applications will depend not only on the biodegradation properties but also on their biocompatibility. For use in medical applications, materials must be biocompatible, which means that they should not cause severe immune reactions when introduced to soft tissues or blood of a host organism during degradation in the body to be considered biocompatible.

PHAs not only appear in microorganisms as storage materials but are also ubiquitous in other natural plants as well as animals, and their metabolism and excretion are both well understood. The monomeric component of P(3HB) and R-3-hydroxybutanoic acid is a product of cell metabolism, produced during fatty acid oxidation in the liver. This hydroxyl acid is a ketone body that is biosynthesized in mitochondria of the liver and is used by the brain as a fuel source. 3-Hydroxybutyric acid is a normal constituent of human blood in concentrations between 0.3 and 1.3 mM [23]. An attractive progress has been made after the finding of the very widespread dispersal of PHB as a low molecular weight oligomer (120–200 monomers) in microorganisms, plants, and animals, including humans. In many cases, this form of PHB is found as a PHB calcium polyphosphate complex in membranes that seems to function as an ion channel through cell membranes [24].

Various medical applications of PHA have been explored extensively in recent years. PHAs have been used to develop devices including nerve repair devices, repair patches, cardiovascular patches, orthopaedic pins, adhesion barriers, guided tissue repair/regeneration devices, nerve guides, tendon repair devices, bone-marrow scaffolds, tissue engineered cardiovascular devices, and wound dressings [25–27]. So far, various tests on animal models have shown polymers, from the PHA family, to be compatible with a range of tissues. Surface properties of PHA films have been shown to be favourable for proliferation and attachment of tissue cells [28, 29], suggesting that PHA is suitable for scaffolding materials in tissue engineering. NIH 3T3 fibroblast cells have been shown to adhere and proliferate on PHA membranes [30]. Also, mesenchymal stem cells were also shown to adhere and proliferate on several PHA substrates, with a terpolymer poly(hydroxybutyrate-*co*-hydroxyvalerate-*co*-hydroxyhexanoate) (P(HB-*co*-HV-*co*-HHx)) [31, 32].

PHA matrices have also been tested for hemocompatibility by inspecting the response of mammalian blood when incubated with polymer films. It was shown that PHB or P (HB-*co*-HV), when in contact with blood, did not affect platelet responses, nor did the polymer activate the complement system. However, the polymer purification procedures had to be followed to significantly reduce the amount of bacterial cell wall material associated with the purified PHA [33, 34].

In evaluating P(3HB) as a potential drug delivery matrix, Korsatko et al. reported no significant differences in cellular growth with mice fibroblasts. Small, low molecular weight, crystalline particles of P(3HB) which represent one of the degradation products are expected to rise *in vivo* from the absorption of P(3HB) [35]. At low concentration, these small P(3HB) particles were found to be well tolerated by macrophages, fibroblasts, Kupffer cells, and hepatocytes. Macrophages, Kupffer cells, and to a lesser extent fibroblasts and osteoblasts were found to phagocytise the small particles of P(3HB) (1–20 *μ*m), and the evidence of biodegradation by macrophages was also found [36].

*In vivo* and *in vitro* biocompatibility of PHB and P(HBV) copolymers has been studied in which the effects of P(HB-*co*-HV) polymers on the growth of CHO (Chinese hamster ovary) cells in culture were monitored over a 60-hour period. The polymers, used as solvent cast films, did not inhibit growth of cells during this period, thereby suggesting good biocompatibility [37]. Juni and Nakano studied the *in vivo* biocompatibility of PHB by injecting microspheres (100 *μ*m) into the rat thigh muscle [38]. Transient acute inflammation was observed which was terminated 7 days after injection. The microspheres were further reported as being encapsulated by connective tissue during a 4-week postinjection study period.

Despite the initial acute inflammation observed in various *in vivo* studies with P(HB-HV) which is probably in response to the trauma of implantation or injection, P(HB-HV) polymers generally showed good *in vitro* and *in vivo* biocompatibility [39].

## 5. Polyhydroxyalkanoates in Drug Delivery

Polyhydroxyalkanoates are generally biodegradable and thermoprocessable, making them attractive as biomaterials for applications in conventional medical devices, drug delivery, and tissue engineering. Biodegradable polymers containing an entrapped drug can be placed in the body, and they are used for localized drug delivery accompanied with the controlled release of a drug over a period of months [40]. Degradation of PHA polymers in the tissues of the host organism offers the possibility of coupling this phenomenon with the release of bioactive compounds, such as antibiotic or antitumor drug.

### 5.1. PHA Particles as Drug Carriers

PHAs are biocompatible and hydrophobic; they can also be turned into films, porous matrices, microcapsules, microspheres, and nanoparticles. Drugs can be entrapped or microencapsulated in a PHA homopolymer or copolymer. Microsphere- or microcapsule-based delivery systems have been extensively used for the delivery of a number of drugs such as anesthetics, antibiotics, anti-inflammatory agents, anticancer agents, hormones, steroids, and vaccines [41, 42].

Use of PHA microspheres as carriers for steroids was reported by Gangrade and Price [43]. PHB and P(3HB-3HV) were used to prepare microspheres containing progesterone as a model drug. The incorporation of progesterone into the microspheres was very efficient, and over 80% of the theoretical content was incorporated. The *in vitro* release was the slowest from a microsphere prepared from a copolymer containing 9% HV, which was less porous than the microspheres prepared from other polymers.

 In the early 1990s, PHAs became candidates for use as drug carriers due to their biodegradability, biocompatibility, and their degradation by surface erosion. The potential use of P(3HB) and P(3HB-*co*-3HV) in drug delivery has been evaluated in a number of studies. PHA may be a potential candidate in treating highly resistant infections, as PHA drug delivery systems showed the ability for provision and maintenance of adequate concentrations of antibiotics at infection sites [44, 45]. PHB, PHBV, and P(3HB-4HB) were shown to be useful in the construction of biodegradable, implantable rods for the local delivery of antibiotics in chronic osteomyelitis therapy [46–48]. When comparing the *in vitro* and *in vivo* releases of the anticancer agent lomustine from PHB and PLA microspheres as potential carriers for drug targeting, it was found that the drug was released faster from the PHB microspheres [49]. Incorporation of ethyl or butyl esters of fatty acids into the PHB microspheres increased the rate of the drug release [50].

Sendil et al. used polyhydroxybutyrate-*co*-hydroxyvalerates (PHBV) of various 3-hydroxyvalerate contents containing antibiotic tetracycline which is known to be effective against many of the periodontal disease-related microorganisms, for the construction of a controlled release system [51]. Tetracycline was loaded in the PHBV microspheres and microcapsules both in its acidic from (TC) and in neutral form (TCN) followed by the analysis of the properties by *in vitro* release studies of the resultant systems. It was observed that release was complete before any signs of degradation were observed.

A study using PHB microspheres demonstrated that release of the antitumor drug rubomycin inhibited proliferative activity of Ehrlich's carcinoma in mice [52]. P(3HB) nanoparticles containing prednisolone were prepared using high-pressure homogenization by Koosha et al. [53]. Biphasic release pattern was observed up to 50% of drug loading, with an initial burst effect followed by slow release of drug, and the completion was achieved in 1 to 2 days. Kawaguchi et al. reported the preparation of microspheres of PHB containing the antitumor drug 2′,3′-diacyl-5-fluoro-2′-deoxyuridine [54]. The PHB sphere showed low toxicity and good compatibility in mice and rats. Recently, the application of PHA as a drug delivery carrier in anticancer study was reported by Lu et al. [55]. A sustained release system of P13 K inhibitor (TGX221) based on PHA nanoparticle was developed and used to block the proliferation of cancer cell lines. TGX221 was gradually released from PHA-based nanoparticles, and the growth of cancer cell lines was significantly slower in cells treated with TGX221 nanoparticles.

**Figure 4:**
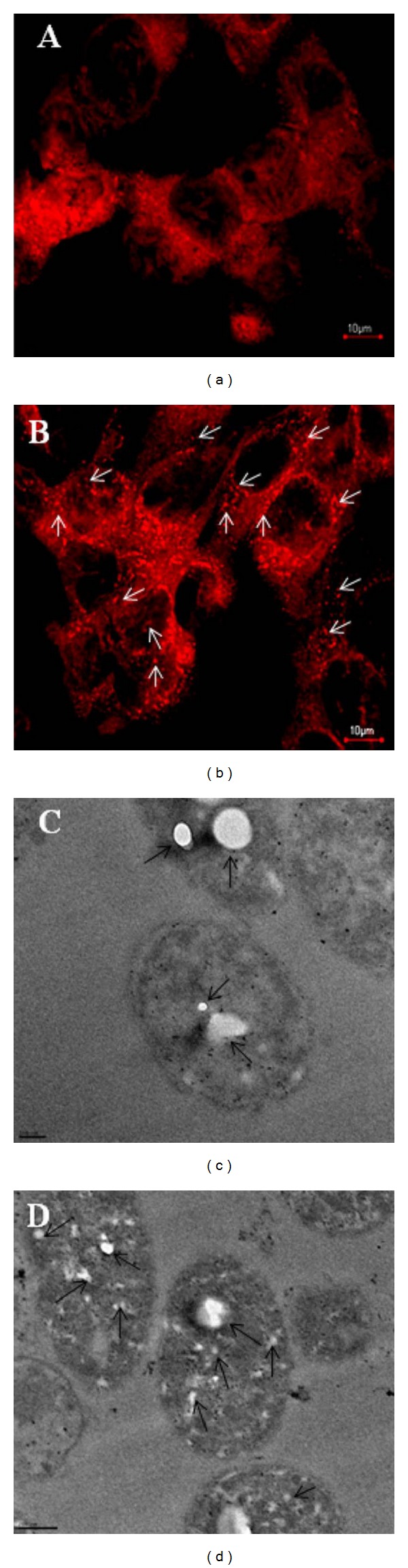
Microscopic study showing the cellular internalization of the P(3HV-*co*-4HB)-*b*-mPEG NPs: (a) CLSM images of the DU145 prostate cancer cells treated with free rhodamine-123 for 12 h; (b) cells treated with rhodamine-loaded NPs for 12 h (scale bar 10 *μ*m); (c) TEM images showing the uptake of NPs in DU145 prostate cancer cells incubated for 6 h (scale bar = 100 nm); (d) cells incubated for 12 h (scale bar = 200 nm). Arrows in the figure represent intracellular localization of NPs. Reprinted with permission from Shah et al. [15], Copyright (2012), Elsevier.

Recently, Shah et al. determined the efficacy and bioavailability of cisplatin, a chemotherapeutic agent used against a variety of tumors, in the form of cisplatin-loaded self-assembled amphiphilic copolymer nanoparticles [15]. Novel amorphous amphiphilic block copolymer P(3HV-*co*-4HB)-*b*-mPEG was synthesized from bacterial copolyester poly(3-hydroxyvalerate-*co*-4-hydroxybutyrate) coupled via transesterification reaction using bis(2-ethyl hexanoate) tin catalyst to monomethoxypoly(ethylene glycol). The *in vitro* release profile of cisplatin from the core hydrophobic domain showed a sustained release of the drug. TEM and confocal microscopy examination revealed clearly the internalization of cisplatin-loaded NPs into the tumor cells (Figure 4) and also revealed a suppression effect by the NPs on tumor cell growth, and they also revealed enhancement of apoptotic process of the tumor cells.

### 5.2. Surface Functionalization of PHA Nanoparticles through Engineered PHA Synthase

**Figure 5:**
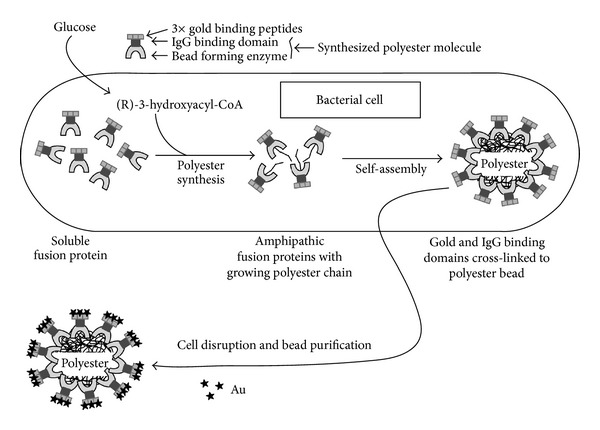
Schematic overview of PHA granules with a multifunctional surface displaying both specific binding sites for certain inorganic substance gold and for IgG. Reprinted with permission from Jahns et al. [58], Copyright (2008), American Chemical Society.

In 2005, Peters and Rehm demonstrated that PHA granule formation was not affected by the fusion of GFP with the N-terminus of the PHA synthase [56]. Further studies were carried out to engineer the PHA synthase as a conjugated form with the enzyme **β**-galactosidase [57]. Conjugated **β**-galactosidase was stable for several months under various storage conditions. This work showed that protein engineering of the PHA synthase to produce functionalized PHA granules could be a useful tool for developing biological particles for various applications. PHA granules were used as biological template structures for molecular biomimetics by Jahns et al. [58]. The PHA synthase was fused to genetically engineered proteins for inorganic surface (GEPIs) and additionally to the ZZ domain of *Staphylococcus aureus*. PHA granules with a multifunctional surface displaying both specific binding sites for certain inorganic substances (gold or silica) and for IgG were produced (Figure 5). These granules may serve as suitable tools for medical imaging procedures when an antibody-mediated targeted delivery of an inorganic contrast agent is desired.

**Figure 6:**
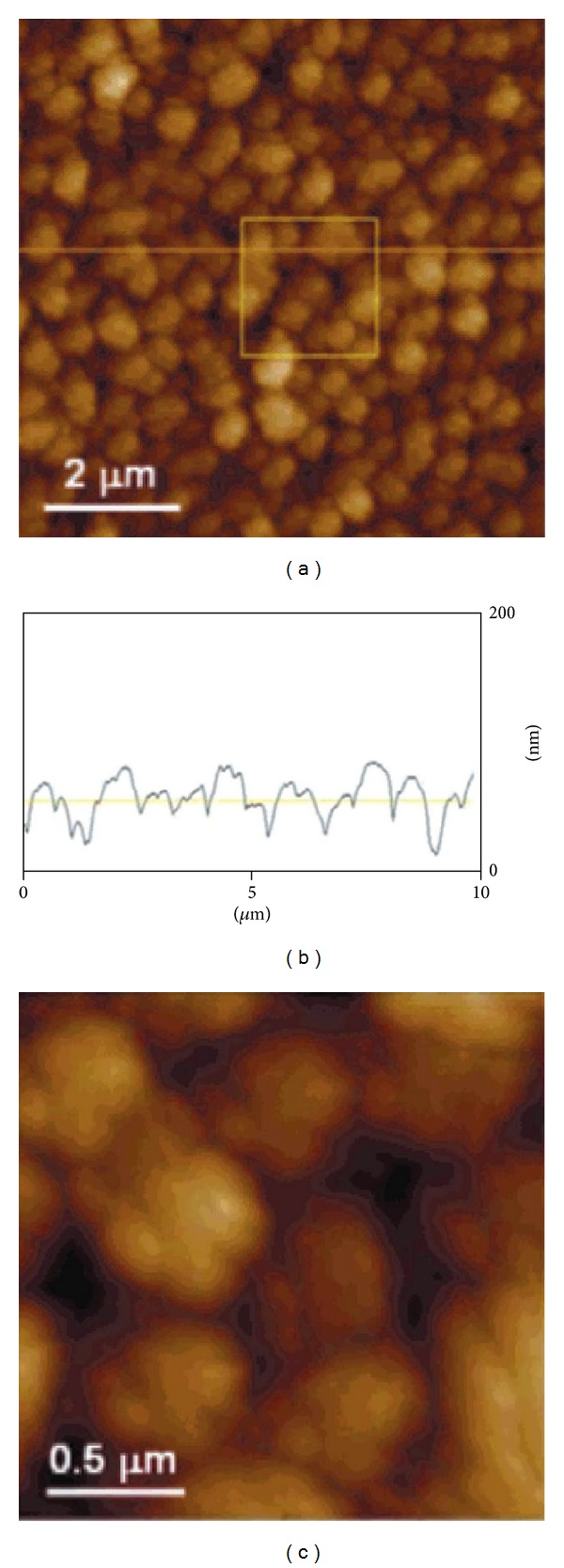
AFM images of the synthesized PHB film on the silicon surface. (a) The cross section (b) taken at the 10 *μ*m horizontal line. Part (c) is the magnified image of the square area (2 *μ*m × 2 *μ*m) in part (a). It shows the microstructure of an individual grain. Reprinted with permission from Kim et al. [59], Copyright (2004), American Chemical Society.

**Figure 7:**
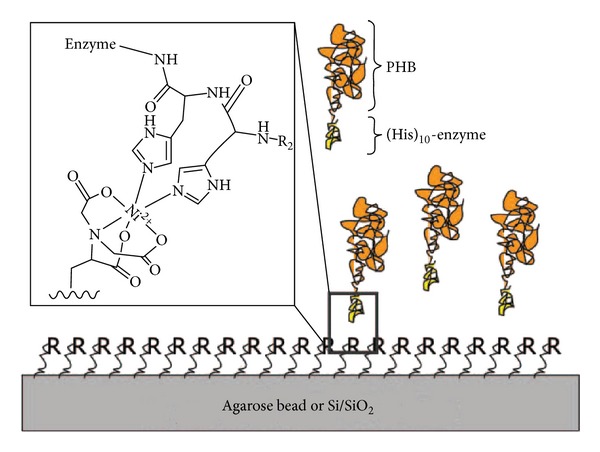
Schematic representation of end-functionalized PHB block copolymers complexing onto an Ni-NTA-derivatized solid surface. The inset shows a part of his-tag complexed with Ni-NTA on the surface. R represents Ni-NTA and R_2_ for the rest of histidine units. Paik et al. (2005) [60]. Reproduced by permission of the Royal Society of Chemistry.

Kim et al. reported a novel system for surface-initiated enzymatic polymerization to modify a solid substrate with biocompatible and biodegradable polymer PHB [59]. Poly-histidine and N-terminus tagged PHA synthase from *Ralstonia eutropha* H16 was used as an initiator for the polymerization through transition metal complexes, Ni^+2^-nitrilotriacetic acid (Ni-NTA) (Figure 6). This system made it possible to initiate polymerization under physiological conditions and, through the specificity of enzyme, tailor the properties of the surface in a highly controlled manner by catalysing the polymerization in the designated area. The modification of solid substrates with PHB could potentially yield polymer-coated implants or controlled release devices for the applications in drug delivery and tissue engineering. Further work by the same group demonstrated a new approach to end functionalization of PHB using genetically engineered PHA synthase and to modify solid surfaces (Figure 7). Modification of PHA end groups by protein engineering aids in the introduction of various functionalities into PHAs which will allow it to interact with specific ligands or receptors. This new approach will be a useful tool to develop novel classes of block copolymers of which one block is a member of the PHA family with potentially 100 different types of monomers, and the other block is a protein with custom-designed sequences and functionalities. Modification of PHA end groups through protein engineering will provide an effective way of introducing diverse functionalities into the biopolymer, which will further broaden its spectrum to other receptors or ligands of interest for advanced delivery systems [60].

### 5.3. PHA Nanoparticles-Based Targeted Drug Delivery

Recently, there is a growing interest in the development of novel drug delivery systems using nanotechnology. Nanoparticles represent a promising drug delivery system of controlled and targeted release and have become an important area of research in the field of drug delivery because they have the ability to deliver a wide range of drugs to various areas of the body for sustained periods of time. The surface properties of Nanoparticles can be modified for targeted drug delivery. A wide variety of drugs can be delivered using nanoparticles via a number of routes. Nanoparticles can be used to deliver hydrophilic drugs, hydrophobic drugs, proteins, vaccines, biological macromolecules, and so forth [64].

Targeted drug delivery systems are designed to deliver drugs at the proper dosage for the required amount of time to a specific site of the body where it is needed, thereby preventing any adverse effects drugs may have on other organs or tissues. Targeted delivery assumes great importance particularly in the case of highly toxic drugs such as chemotherapeutic drugs and highly active and fragile biotechnological molecules such as peptides and proteins. It is widely believed that active targeting, through the modification of nanoparticles with ligands, has the potential to enhance the therapeutic efficacy and reduce the side effects relative to conventional therapeutics [65]. In cancer therapy, the presence of targeting ligands can greatly enhance the retention and cellular uptake of nanoparticles via receptor-mediated endocytosis even though tumor accumulation is largely determined by the physicochemical properties of nanoparticles [66]. This can then lead to higher intracellular drug concentration and increase therapeutic activity, which is particularly important for bioactive macromolecules (e.g., DNA and siRNA) that require intracellular delivery for bioactivity [67].

Microspheres of PHB for the targeted delivery of formalinized vaccine of Staphylococcal enterotoxin B, to the gut-associated lymphoid tissues, were reported by Eldridge et al. [68]. Tissue penetration was specific to Peyer's patches for microspheres of 10 *μ*m or less in diameter. In addition, the PHB microspheres exhibited very good absorption.

**Figure 8:**
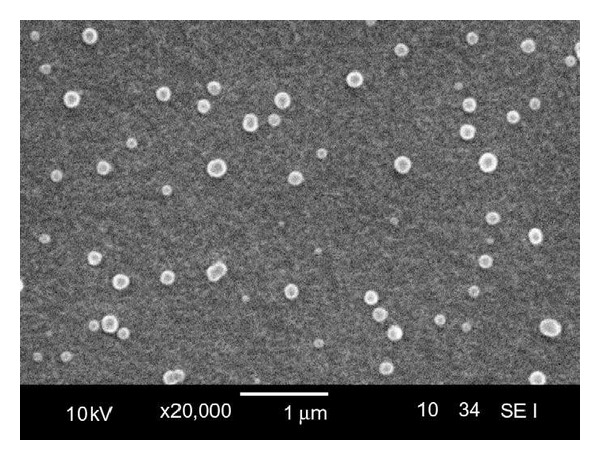
SEM image of RBITC loaded PHA nanoparticles. Reprinted with permission from Yao et al. [61], Copyright (2008), Elsevier.

Yao et al. developed a receptor-mediated drug delivery system in which rhodamine B isothiocyanate (RBITC) model drug was targeted to cancer cells or macrophages by incorporating with P(HB-*co*-HHx) and associating with a recombinant PhaP phasin protein from *C. necator*. SEM image of RBITC-loaded nanoparticles is shown in Figure 8 [61]. These recombinant phasins were fused to ligands mannosylated human *α*1 acid glycoprotein (hAGP) and human epidermal growth factor (hEGF) for targeting cancer cells or macrophages, respectively. hAGP is recognized by receptors on macrophages, and hEGF is recognized by receptors on hepatocellular carcinoma cells. Proper targeting of PHA/RBITC nanoparticles to each cell line was demonstrated by fluorescence microscopy which showed that the nanoparticles were directed to the correct type of tissue by intake through correct type of cell, proving targeted delivery.

Biopolymer-based nanocarriers with targeting capability for imaging and drug delivery to tumors through molecular recognition of the cancer specific marker, integrin, were developed by Kim et al. [69]. The Arg-Gly-Asp (RGD) motif was used as a ligand to target *α*_*v*_ *β*_3_ integrins, which have been identified as cell surface receptors that mediate the adhesion of cells to the extracellular matrix and are highly expressed in various cancer cells. PHA synthase was fused to RGD-containing peptide through protein engineering, and further expression in recombinant *E. coli* was done, followed by purification. The engineered enzyme was used to produce an amphiphilic protein-polymer hybrid with a specific end functionality. The resulting block copolymer with RGD peptide at one end was readily self-assembled into a micellar structure in the presence of substrate 3HB-CoA and was successfully used to target tumor cells.

**Figure 9:**
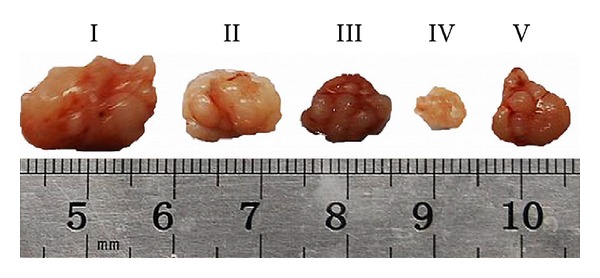
Representative photographs of each group's tumor. I: normal saline; II: free DOX; III: DOX/P(HB-HO) NPs; IV: DOX/FA-PEG-P(HB-HO) NPs; V: DOX/FA-PEG-P(HB-HO) NPs + 1 mM free folic acid. Reprinted with permission from Zhang et al. [62], Copyright (2010), Elsevier.

Zhang et al. developed a novel targeting drug delivery system using poly(3-hydroxybutyrate-*co*-3-hydroxyoctanoate) [P(HB-HO)] as the drug carrier, folic acid (FA) as the targeting ligand, and doxorubicin (DOX) as the model anticancer drug [62]. The average size, drug loading capacity, and encapsulation efficiency of the prepared DOX-loaded, folate-mediated P(HB-HO) nanoparticles (DOX/FA-PEG-P(HB-HO) NPs) were found to be around 240 nm, 29.6%, and 83.5%, respectively. The intracellular uptake tests of the nanoparticles (NPs) *in vitro* showed that the DOX/FA-PEG-P(HB-HO) NPs were more efficiently taken up by HeLa cells. In addition, DOX/FA-PEG-P(HB-HO) NPs (IC_50_ = 0.87 *μ*M) showed greater cytotoxicity to HeLa cells than the other treated groups. *In vivo* antitumor activity of the DOX/FA-PEG-P(HB-HO) NPs showed a much better therapeutic efficacy in inhibiting tumor growth, and the final mean tumor volume was 178.91 ± 17.43 mm^3^, significantly smaller than normal saline control group (542.58 ± 45.19 mm^3^) (Figure 9) which shows that these NPs are effective in selective delivery of anticancer drug to the folate receptor-overexpressed cancer cells.

**Figure 10:**
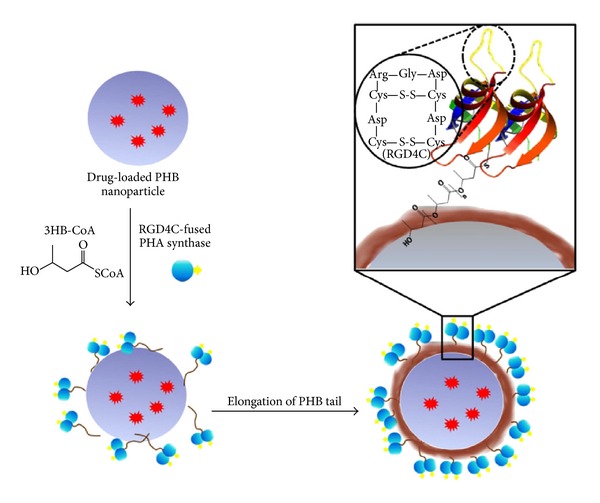
Surface functionalization of hydrophobic PHB nanoparticles through enzymatic reaction. Reprinted with permission from Lee et al. [63], Copyright (2011), Elsevier.

Recently, Lee et al. demonstrated a new approach to prepare a nanocarrier system with targeting capability for imaging and drug delivery to cancer cells by integrating the unique catalytic characteristics of PHA synthase with simple oil into water emulsion methods (Figure 10) [63]. The effective coupling between the hydrophobic surface of PHB nanoparticle and PHB chain grown from the enzyme fused with a specific ligand provided a simple way of functionalizing nanoparticle with active protein layers in aqueous environment. The surface of nanoparticles was functionalized with tumor-specific ligand, RGD4C, fused with PHA synthase. The functionalized PHB nanoparticles showed a specific affinity to MDA-MB 231 breast cancer cells indicating that the tumor-specific ligand, RGD4C, was effectively displayed on the surface of PHB nanoparticles through enzymatic modification and confered targeting capability on the drug carrier.

## 6. Conclusion

Increased interest in the use of PHA for medical applications had arisen the response to the need for the emerging field of drug delivery, where a much wider range of biodegradable and biocompatible polymers are being sought for use as drug carriers. Because of their versatility and wide range of properties, biodegradable PHAs are being used as novel drug delivery systems. In particular, PHA-based drug carrier holds tremendous promise in the areas of cancer therapy and controlled delivery of drugs including steroids, vaccines, and other biological molecules. They can be formulated for targeted drug delivery to tumours or organs. Various successful studies using PHA as a drug carrier have clearly demonstrated that PHA possesses biodegradability and biocompatibility for drug carrier use. PHA has a wide variety of applications, among which the medical applications seem to be the most economically practical area. With the currently increased interest level and the extensive research being carried out in this area, PHAs are potentially emerging as environmentally friendly materials of the next generation with a wide range of applicability.
